# Formation of chlorate and perchlorate during electrochemical oxidation by Magnéli phase Ti_4_O_7_ anode: inhibitory effects of coexisting constituents

**DOI:** 10.1038/s41598-022-19310-5

**Published:** 2022-09-23

**Authors:** Lu Wang, Yaye Wang, Yufei Sui, Junhe Lu, Baowei Hu, Qingguo Huang

**Affiliations:** 1 School of Life Science, Shaoxing University, Shaoxing, 312000 China; 2 Department of Environmental Science and Engineering, Nanjing Agricultural University, Nanjing, 210095 China; 3 Department of Crop and Soil Sciences, University of Georgia, Griffin, GA 30223 USA

**Keywords:** Environmental chemistry, Pollution remediation

## Abstract

Formation of chlorate (ClO_3_^−^) and perchlorate (ClO_4_^−^) as by-products in electrooxidation process has raised concern. In the present study, the formation of ClO_3_^−^ and ClO_4_^−^ in the presence of 1.0 mM Cl^−^ on boron doped diamond (BDD) and Magneli phase titanium suboxide (Ti_4_O_7_) anodes were evaluated. The Cl^−^ was transformed to ClO_3_^−^ (temporal maximum 276.2 μM) in the first 0.5 h on BDD anodes with a constant current density of 10 mA cm^2^, while approximately 1000 μM ClO_4_^−^ was formed after 4.0 h. The formation of ClO_3_^−^ on the Ti_4_O_7_ anode was slower, reaching a temporary maximum of approximately 350.6 μM in 4.0 h, and the formation of ClO_4_^−^ was also slower on the Ti_4_O_7_ anode, taking 8.0 h to reach 780.0 μM. Compared with the BDD anode, the rate of ClO_3_^−^ and ClO_4_^−^ formation on the Ti_4_O_7_ anode were always slower, regardless of the supporting electrolytes used in the experiments, including Na_2_SO_4_, NaNO_3_, Na_2_B_4_O_7_, and Na_2_HPO_4_. It is interesting that the formation of ClO_4_^−^ during electrooxidation was largely mitigated or even eliminated, when methanol, KI, and H_2_O_2_ were included in the reaction solutions. The mechanism of the inhibition on Cl^−^ transformation by electrooxidation was explored.

## Introduction

Electrooxidation (EO) process is a promising technology in wastewater treatment^1–4^. EO process has been demonstrated to be a viable means to decompose a broad spectrum of recalcitrant organic pollutants that are not removable by conventional treatment processes, including pharmaceuticals, endocrine disruptors, phenolic compounds, and particularly per- and polyfluoroalkyl substances (PFASs)^5–9^. EO is a chemical destructive technology that promotes organic pollutants degradation by direct electron transfer from organic contaminants to the anode and attack by hydroxyl free radicals and other reactive oxygen species that are also generated on the anode surfaces during the EO process^10^.

Sufficiently stable and effective anode materials for EO water treatment have been developed in the last decades, including mixed oxides, such as iridium and/or ruthenium oxides^11–13^, titanium dioxide^14^, and doped diamond electrodes (BDD)^15–17^. This is one of the important reasons why the EO process has approached to technical maturity only recently^18^. Magnéli phase titanium sub-oxides, such as Ti_4_O_7_, have recently been explored as promising electrode materials for EO applications because of their high conductivity and chemical inertness. Ti_4_O_7_ anodes have been shown to oxidize recalcitrant contaminants by a combination of direct electron transfer (DET) and indirect reactions with HO^·^ produced at the anode surface from water oxidation^10^. Our recent studies have demonstrated the degradation and mineralization of Perfluorooctanesulfonate (PFOS, the one most commonly used per-fluoroalkyl acids) on the Magneli phase Ti_4_O_7_ anode^19,20^.

One factor limiting the application of EO in water/wastewater treatment is that its strongly oxidizing conditions also result in the formation of toxic by-products in the presence of Cl^−^, such as chlorate (ClO_3_^−^) and perchlorate (ClO_4_^−^). In particular, ClO_4_^−^ is difficult to remove from water and its consumption has been linked to health risks associated with disruption of the endocrine and reproductive systems^21^. These risks have caused the U.S. Environmental Protection Agency (EPA) to regulate perchlorate under the Safe Drinking Water Act, although an established federal limit has not yet been set^22^. The formation of ClO_4_^−^ was reported during EO using several anode materials (e.g., BDD and Ti_4_O_7_)^23^. The presence of Cl^−^ lead to the formation of free chlorine (HOCl) that is further converted to ClO_3_^−^ and ClO_4_^−^ in EO systems using both BDD and Ti_4_O_7_ anodes. This transformation process appeared much faster on BDD than Ti_4_O_7_ anode^24^. It is desirable to develop electrooxidation systems that minimize the formation of chlorine-related toxic by-products for water/wastewater treatment applications.

The purpose of this study was to systematically investigate the formation of ClO_3_^−^ and ClO_4_^−^ in solutions containing Cl^−^ during electrooxidation on Magneli phase Ti_4_O_7_ anode and compares it to those on BDD anode. The experiments were performed in different supporting electrolytes at different electrochemical conditions. The effects of a few co-existing constituents were assessed to investigate the inhibition of ClO_3_^−^ and ClO_4_^−^ formation on the Ti_4_O_7_ anode. The findings provide a basis for devising strategies to reduce the formation of ClO_3_^−^ and ClO_4_^−^ in EO on Ti_4_O_7_ anode.

## Materials and methods

### Reagents and materials

All chemicals used in the experiments were of reagent grade or higher. ClO_3_^−^ was purchased from Sigma-Aldrich (St. Louis, MO). ClO_4_^−^, NaCl, and HPLC grade methanol (MeOH) were obtained from Fisher Chemical. Na_2_SO_4_, NaNO_3_, Na_2_B_4_O_7_ Na_2_HPO_4_, NaH_2_PO_4_, H_3_PO_4_, H_2_O_2_, and KI were supplied by J.T. Baker. All stock solutions were prepared in ultrapure water (18.2 MΩ cm^−1^) produced by a Barnstead Nano pure water purification system.

### Experimental procedures

EO experiment was carried out in an undivided rectangular cell (10 cm × 5 cm × 2.5 cm) made of acrylic materials. A ceramic plate Ti_4_O_7_ (10 cm × 5 cm) or a Si/BDD plate of the same size (both sides coated, NeoCoat, Switzerland) was used as the anode, and two 304 stainless steel plates of the same size as the anode, placed on both sides of the anode in parallel with an interval of about 2.5 cm, were used as the cathodes. The Ti_4_O_7_ electrodes were fabricated according to the method used in our previous study^20,24^, and information on their preparation and characterization is described in detail in Supporting Information (Text S1). During each experiment, the electrolytic cell contained a 200 mL solution containing Cl^−^ (1.0 mM) and Na_2_SO_4_ (100 mM) or other salts (NaNO_3_, Na_2_B_4_O_7_, Na_2_HPO_4_, NaH_2_PO_4_, H_3_PO_4_) as supporting electrolytes stirred at 700 rpm unless otherwise specified. Some EO experiments were performed to explore the impact of pH with Na_2_HPO_4_ electrolyte as a buffer for pH 10–11, NaH_2_PO_4_ + Na_2_HPO_4_ for pH 6–7, and H_3_PO_4_ for pH 2–3. In some EO experiments, MeOH (10–1000 mM), KI (20–100 mM) or H_2_O_2_ (100–1000 mM) were spiked to the electrolyte solution to explore their impact on the formation of chlorate and perchlorate. All EO experiments were conducted at room temperature.

A constant electric current was supplied at the of 10 mA cm^−2^ density using a controllable DC power source (Electro Industries Inc., Monticello, MN), unless otherwise specified. The submerged surface area on both sides of the anode (total geometric surface area was 78 cm^2^) was used for calculating the electric current density. A CHI 660E electrochemical workstation (CH Instruments, Inc., Austin, TX) was used to measure the anodic potential using an Ag/AgCl reference electrode placed close to the anode, with the potential drop in solution (iRs) compensated. Triplicate samples (1.0 mL each) were withdrawn at pre-selected time points, with the power source paused and the solution continuously stirred to ensure homogeneity. The samples were stored at 4 °C until further analysis. The data were plotted with error bars representing the maximum and minimum of duplicated test results. The temperature of solution was monitored and no significant change was found during electrolysis process.

### Analysis methods

Free chlorine, ClO_3_^−^, and ClO_4_^−^ were quantified in selected samples. The Concentration of HClO was measured by spectrophotometer at 510 nm (Beckman Coulter DU 800, Brea, CA). A 2.5-mL aliquot sample was immediately mixed with 0.25-mL DPD solution (8.0 mM). DPD is oxidized by HOCl to show a red color. ClO_3_^−^ and ClO_4_^−^ were analyzed using a Waters (Milford, MA) ultra-high performance liquid chromatography with an electrospray ionization (ESI) source (UPLC-MS/MS). Detailed UPLC-MS/MS analytical parameters can be found in Text S2. Quantification of the ClO_3_^−^ and ClO_4_^−^ was based on multipoint standard calibration.

## Results and discussion

### Formation of ClO_3_^−^ and ClO_4_^−^ in EO systems

**Figure 1:**
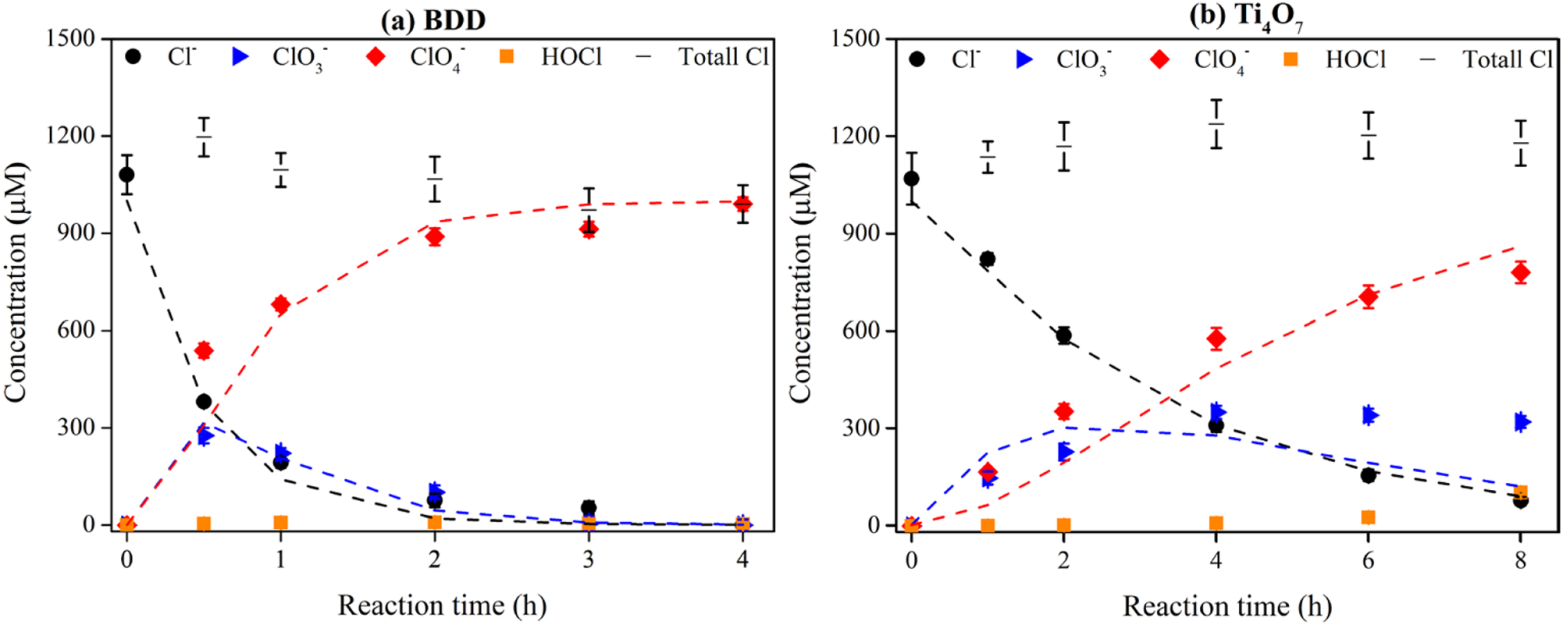
Comparison of chlorine species during the electrochemical oxidation of Cl^−^ on different anodes. Dashed lines show simulated results. Conditions: [Cl^−^]_0_ = 1.0 mM, [Na_2_SO_4_] = 100 mM, current density = 10 mA cm^-2^.

Cl^−^ can be oxidized in EO systems to form reactive chlorine species that lead to ClO_3_^−^ and ClO_4_^−^. It was shown that the presence of 1.0-mM Cl^−^ resulted in increased HOCl, ClO_3_^−^, and ClO_4_^−^ on BDD and Ti_4_O_7_ anodes (Fig. 1). Almost no appreciable HOCl was detected during the 4.0 h electrooxidation process on the BDD anode, while the concentration of HOCl increased continuously in the system with Ti_4_O_7_ anode and reached 103.2 μM at 8.0 h. In both systems, ClO_3_^−^ concentration increased and then plateaued, while the concentration of ClO_4_^−^ increased continuously. The transformation of Cl^−^ was faster on BDD anode in general. The data (Fig. 1) indicate that the concentration of ClO_3_^−^ reached 276.2 μM in the first 0.5 h and then decreased on BDD anode, while almost all Cl (about 1000 μM) was transformed to ClO_4_^−^ within 4 h. The formation of ClO_3_^−^ on Ti_4_O_7_ electrode was slower, reaching a plateau of ~ 350.6 μM in 4.0 h and then decreasing slowly. The formation of ClO_4_^−^ also appeared to be more slowly on Ti_4_O_7_ electrode, taking about 8.0 h to reach 780.0 μM.

Cl^−^ can be transformed in electrooxidation by direct electron transfer (DET) to ClO_3_^−^ and ClO_4_^−^ through a pathway of multiple steps (R1–R3). Direct oxidation of Cl^−^ on BDD electrode generated Cl^·^ and hypochlorite. However, unlike BDD electrode, the oxidation of Cl^−^ due to DET on Ti_4_O_7_ anode was not as effective, thus resulting in slower formation of ClO_3_^−^ and ClO_4_^−^ than on BDD anode^24^. Note that indirect routes (R4–R5) can lead to Cl^−^ generation on both Ti_4_O_7_ and BDD anode, which can further go through the reactions in R2 and R3 to form HOCl and chlorinated by-products. The conversion of Cl^−^ to HOCl and the chlorinated byproducts via both DET and indirect routes involves the hydroxyl radicals (HO^−^) that are formed by water oxidation on anode.

R1 $$\equiv \mathrm{S}+{\mathrm{Cl}}^{-}\to \equiv \mathrm{S}\left({\mathrm{Cl}}_{\mathrm{ads}}^{\cdot }\right)+{\mathrm{e}}^{-}$$

R2 $${\text{C}}{\text{l}}^{\cdot }+{\text{H}}{\text{O}}^{\cdot }\stackrel{-{\text{H}}^{+}}{\to }{\text{OC}}{\text{l}}^{-}$$

R3 $${\text{OC}}{\text{l}}^{-}\stackrel{\text{-e,}+{\text{H}}{\text{O}}^{\cdot },-{\text{H}}^{+}\hspace{1em}}{\to }{\text{Cl}}{\text{O}}_{2}^{-}\stackrel{\text{-e,}+\text{ H}{\text{O}}^{\cdot },-{\text{H}}^{+}\hspace{1em}}{\to }{\text{Cl}}{\text{O}}_{3}^{-}\stackrel{\text{-e, }+{\text{H}}{\text{O}}^{\cdot },-{\text{H}}^{+}}{\to }{\text{Cl}}{\text{O}}_{4}^{-}$$

R4 $${\text{HO}}^{\cdot }+{\text{Cl}}^{-}\leftrightarrow {\text{ClHO}}^{\cdot -}\quad k = 4.3 \times 10^{9}\,{\mathrm{M}}^{-1}\,{\mathrm{s}}^{-1}$$

R5 $${\text{ClHO}}^{\cdot -}+{\mathrm{H}}^{+}\to {\text{Cl}}^{\cdot }+{\mathrm{H}}_{2}{\mathrm{O}}\quad k = 2.1 \times 10^{10}\,{\mathrm{M}}^{-1} \, {\mathrm{s}}^{-1}$$

The rate of Cl^−^ conversion to chlorate and perchlorate in EO systems has been simulated using a model of two sequential steps by assuming each step as pseudo-first-order kinetics (R6–R7)^23,25^. The rate constants *k*_*1*_ and *k*_*2*_ in such sequential equations were obtained by fitting the data as shown in Fig. 1 a and b using the software *Kintecus* v6.80^26^. The values of *k*_*1*_ and *k*_*2*_ were fitted to be 5.40 × 10^−4^ and 7.16 × 10^−4^ s^−1^, respectively, on the BDD anode, while for Ti_4_O_7_ anode the values were 8.59 × 10^−5^ and 1.34 × 10^−4^ s^−1^, respectively. This indicates that Cl^−^ is oxidized to ClO_3_^−^ and ClO_4_^−^ more easily on BDD, evidenced by the larger *k*_*1*_ and *k*_*2*_ on the BDD anode than on Ti_4_O_7_ anode. Retarded formation of ClO_3_^−^ and ClO_4_^−^ makes it advantageous to apply Ti_4_O_7_ anodes in water/wastewater treatment.

R6 $${\mathrm{Cl}}^{-}\stackrel{{\mathrm{HO}}^{\cdot }}{\to }{\mathrm{ClO}}_{3}^{-} \quad k_1 = -\frac{{dc}_{C{l}^{-}}}{dt}$$

R7 $${\mathrm{ClO}}_{3}^{-}\stackrel{{\mathrm{HO}}^{\cdot }}{\to }{\mathrm{ClO}}_{4}^{-} \quad k_2 = -\frac{{dc}_{Cl{O}_{3}^{-}}}{dt}$$

### Effects of electrolytes on the formation of ClO_3_^−^ and ClO_4_^−^

**Figure 2:**
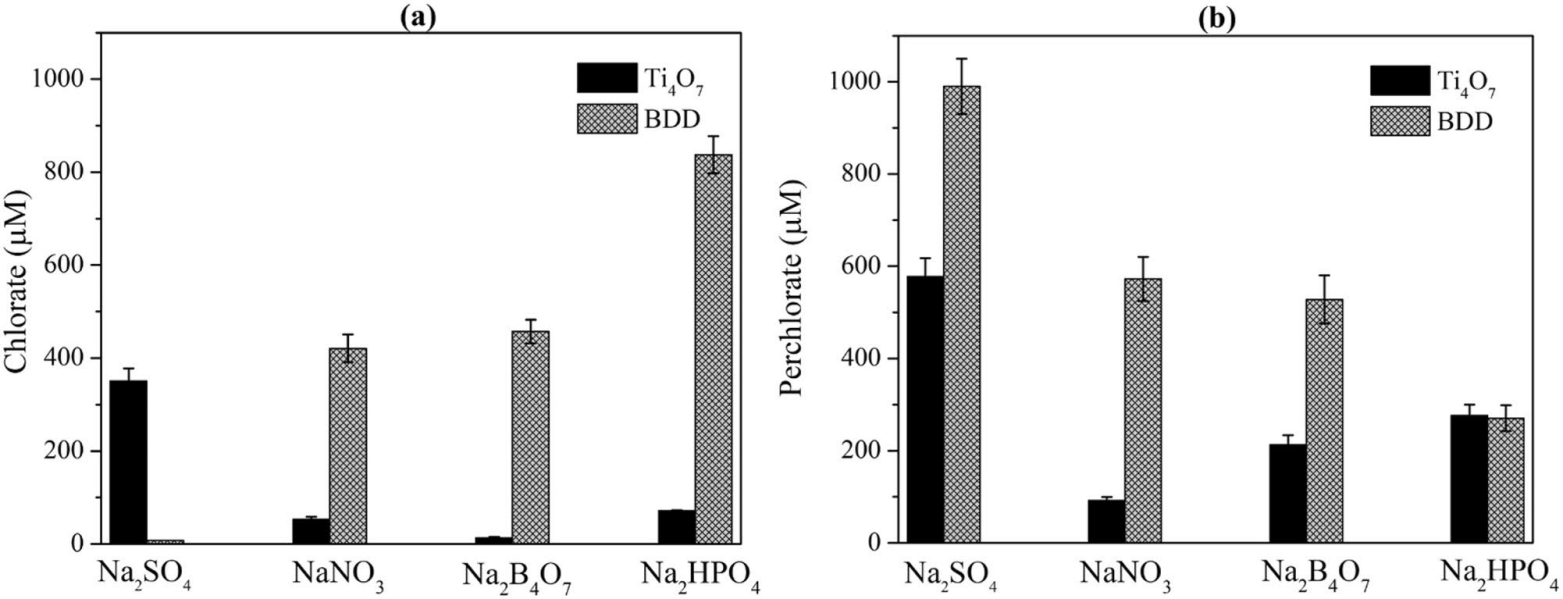
Comparison of ClO_3_^−^ and ClO_4_^−^ formation by the electrochemical oxidation with Cl^−^ in different electrolyte solutions. [Cl^−^]_0_ = 1.0 mM, electrolyte concentration = 100 mM, current density = 10 mA cm^-2^, reaction time = 4.0 h.

A set of experiments were performed to evaluate the ClO_3_^−^ and ClO_4_^−^ formation by EO with BDD and Ti_4_O_7_ anode in solutions containing different supporting electrolytes, including 100-mM Na_2_SO_4_, NaNO_3_, Na_2_B_4_O_7_, and Na_2_HPO_4_. The concentrations of ClO_3_^−^ and ClO_4_^−^ measured in different electrolyte solutions are summarized in Fig. 2. As shown in Fig. 2a and b, ClO_4_^−^ concentration reached 990 μM after 4.0 h with BDD anode and Na_2_SO_4_ as supporting electrolyte, which accounts for about 99% of the total Cl^−^ initially included in the solution. Almost no ClO_3_^−^ was detected. At the same current density, the formation of ClO_4_^−^ was slower with NaNO_3_, Na_2_B_4_O_7_, and Na_2_HPO_4_ as supporting electrolytes on the BDD electrode. It is known that sulfate radical (SO_4_^·−^) can be formed by one-electron oxidation of sulfate ion at the anode, which can participate in the oxidation of organics^15^ and chloride^27^. Occurrence of peroxodiphosphate was observed during the electrolysis of solutions containing phosphate with BDD anodes^28^. Hence, there could be competitive oxidation reactions from phosphate, although peroxodisulphate was also formed in sulfate containing solution^29^. The use of NaNO_3_ as an electrolyte can promote the formation of ammonium and other reduced nitrogen species by electrochemical reduction^30^. Ammonium can react with free chlorine, favoring the formation of chloramines and reducing the potential formation of chlorate and perchlorate^31–33^.

Overall, the transformation was more rapid on BDD anode in all the supporting electrolyte solutions. As shown in Fig. 2, the total ClO_3_^−^ and ClO_4_^−^ concentration was lower when Ti_4_O_7_ was used as the anode. For example, the ClO_4_^−^ concentrations were 572.62 and 527.92 μM, respectively, after 4.0 h on the BDD anode with NaNO_3_ and Na_2_B_4_O_7_ as supporting electrolytes, while on Ti_4_O_7_ anode, they were 92.37 and 212.84 μM, respectively. In particular, the ClO_4_^−^ concentration in BDD system was 572.6 μM after 4.0 h with NaNO_3_ as the supporting electrolyte, while it was only 92.4 μM at the same condition on the Ti_4_O_7_ anode.

### Inhibitory effect of co-existing constituents

Experiments were performed to examine EO in the presence of Cl^−^ as well as a few co-existing constituents, including MeOH, H_2_O_2_ and KI, so as to investigate the effect of the coexisting constituents on the formation of ClO_3_^−^ and ClO_4_^−^ with Ti_4_O_7_ anode.

#### MeOH

**Figure 3:**
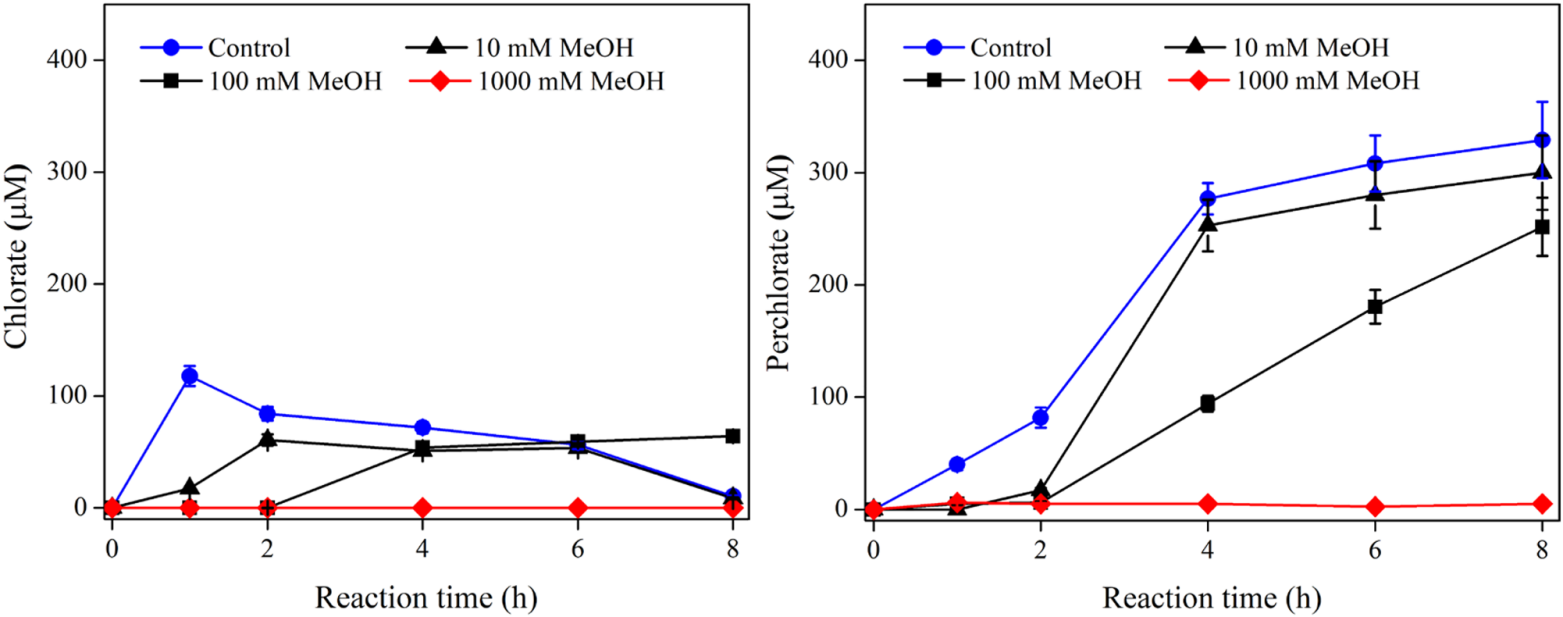
Concentration of ClO_3_^−^ and ClO_4_^−^ during the electrochemical oxidation of Cl^−^ in the presence of MeOH on Ti_4_O_7_ anodes. Conditions: [Cl^−^]_0_ = 1.0 mM, [Na_2_HPO_4_] = 100 mM, current density = 10 mA cm^-2^.

Ion exchange resin (IXR) exchange/adsorption has been shown effective to remove PFAS from water. Regeneration of PFAS-laden IXR generates a low-volume, high-concentration liquid waste known as still bottoms that contains high concentrations of PFASs, salts, and residual organic content, including MeOH that is often used as organic co-solvent for IXR regeneration. Our recent studies showed that the MeOH content in still bottoms may play a role in chloride oxidation^20^. In this section, we designed experiments to further explore the effects of MeOH during the transformation of Cl^−^ by EO. As such, the EO experiment was performed in 100-mM Na_2_HPO_4_ solutions containing 1.0 mM Cl^−^ and varying quantities of MeOH. The addition of MeOH appeared to impact the conductivity of the reaction solution slightly. The conductivity dropped from 10.51 mS cm^−1^ to 9.79 mS cm^−1^, but the anodic potential increased at the same current density (10 mA cm^2^) (Fig. S1a), from 2.93 V in the absence of MeOH increasing to 3.22 V with 100 mM MeOH. The presence of MeOH decreased the conductivity of the solution, and thus anodic potential increased at the same current density. The formation of ClO_3_^−^ and ClO_4_^−^ during EO treatment at 10 mA cm^−2^ is displayed in Fig. 3. In the absence of MeOH, ClO_3_^−^ reached 117.8 μM in about 1.0 h and then decreased. The value decreased to 17.3 and 0.0 μM containing 10 mM and 100 mM MeOH, respectively. Such a time course profile indicates the further reaction of ClO_3_^−^. The formation of ClO_4_^−^ increased monotonically, reaching 329.0 μM in 8.0 h in the absence of MeOH. When 10 mM and 100 mM MeOH were spiked, almost no ClO_4_^−^ were formed for the first 2.0 h, after which ClO_4_^−^ started to increase, reaching 300.0 μM and 251.8 μM in 8.0 h, respectively. The formation of ClO_4_^−^ was completely inhibited when 1000 mM MeOH was added, indicating that MeOH inhibited the formation of ClO_3_^−^ and ClO_4_^−^. Delayed formation of ClO_4_^−^ in the presence of lower MeOH dosage (10 and 100 mM) may be caused by MeOH depletion over time. Formation of ClO_3_^−^ and ClO_4_^−^ was neither observed in acid or neutral conditions when 1000-mM MeOH was spiked, by respectively using 50-mM NaH_2_PO_4_ + 50-mM Na_2_HPO_4_ (pH 6–7) or 100-mM H_3_PO_4 (_pH 2–3) as electrolytes instead of Na_2_HPO_4_ (pH 10–11).

A prior study proved that Cl^−^ was not oxidized to Cl^·^ via DET on the Ti_4_O_7_ anode, while Cl^·^ was formed mainly through the indirect pathways (R4−R5)^24^. Cl^·^ reacts with another Cl^−^ to form Cl_2_^·−^. Cl^·^ and Cl_2_^·−^ also combine with each other to form free chlorine (Cl_2_, HClO)^21,34,35^. These reactive chlorine species may accumulate and diffuse away from the anode surface, and finally convert into ClO_3_^−^ and ClO_4_^−^. MeOH can transform HO^·^ into perhydroxyl radicals (with a second-order rate constant is 2.1 × 10^9^ M^−1^ s^−1^). Meanwhile, the reaction rate constant between MeOH and Cl^·^ is 5.7 × 10^9^ M^−1^ s^−1^. MeOH could consume Cl^·^ in the bulk solution and HO^·^ (if present). The formation of ClO_3_^−^ and ClO_4_^−^ was thus reduced with low concentrations of MeOH, while a high concentration of MeOH can rapidly transform Cl^·^, inhibiting the generation of ClO_3_^−^ and ClO_4_^−^.

#### H_2_O_2_

**Figure 4:**
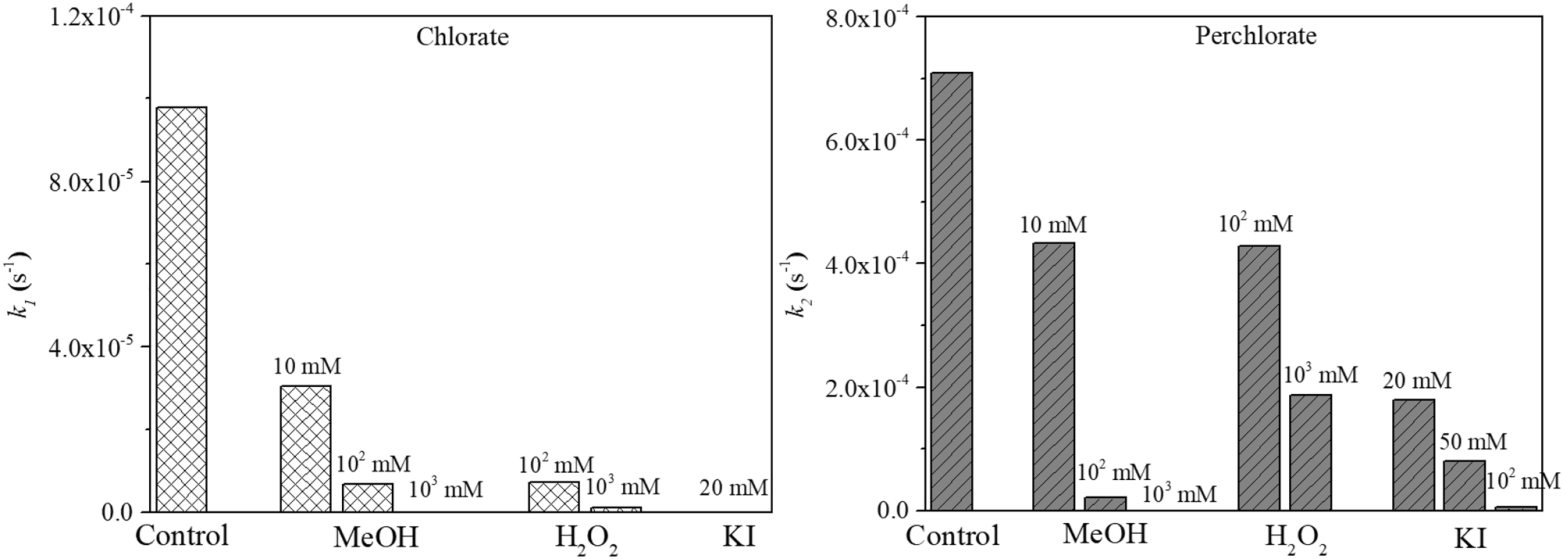
Comparison of fitted *k*_*1*_ (R6) and *k*_*2*_ (R7) during the electrochemical oxidation with the addition of MeOH, H_2_O_2_, and KI.

Yang et al. found that the formation of ClO_4_^−^ during EO with BDD anode can be largely inhibited by adding H_2_O_2_^23^. Therefore, H_2_O_2_, a commonly used quenchers were also investigated in this study. The time-course data of ClO_3_^−^ and ClO_4_^−^ formation in the presence of H_2_O_2_ are shown in Fig. S2. Using Kintecus v6.80, the data in Fig. S2 were fit to obtain *k*_*1*_ and *k*_*2*_ represented in equation R6−R7, and they were 9.78 × 10^–5^ and 7.09 × 10^–4^ s^-1^, respectively, in the absence of co-existing constituents (Fig. 4). The values of *k*_*1*_ and *k*_*2*_ decreased to 1.16 × 10^–6^ and 1.87 × 10^–4^ s^-1^ when 1000-mM H_2_O_2_ were spiked, respectively. The data shown in Fig. S2 and Fig. 4 also showed that addition of H_2_O_2_ at 1000 mM also significantly limited ClO_3_^−^ and ClO_4_^−^ formation during the EO.

H_2_O_2_ is known to be both an oxidant (H_2_O_2_/H_2_O, *E*^*0*^ = 1.76 V) and a reductant (O_2_/H_2_O_2_, *E*^*0*^ = 0.68 V) depending on the composition of the reaction media. Thus, Earlier studies have demonstrated that HOCl can be reduced back to Cl^−^ by H_2_O_2_^36,37^ (R8–R9). In addition to free chlorine, H_2_O_2_ can also react with the chlorine radical species directly (R10-R11). Thus, it is presumed that the reduction of HOCl and chlorine radical species by H_2_O_2_ outweighed the oxidation of Cl^−^ by H_2_O_2_ in the EO system, and thus decreased ClO_3_^−^ and ClO_4_^−^ formation. Moreover, H_2_O_2_ may react with ClO_3_^−^ to form chlorine dioxide (R12)^38,39^, thus further reducing the formation of ClO_3_^−^.

R8 $$\mathrm{HOCl}+{\mathrm{H}}_{2}{\mathrm{O}}_{2}\to {\mathrm{H}}^{+}+{\mathrm{Cl}}^{-}+{\mathrm{H}}_{2}\mathrm{O}+{\mathrm{O}}_{2}\quad k = 1.1 \times 10^4\, \mathrm{ M}^{-1}\mathrm{s}^{-1}$$

R9 $${\mathrm{Cl}}_{2}+{\mathrm{H}}_{2}{\mathrm{O}}_{2}\to {\mathrm{O}}_{2}+2\mathrm{HCl }\quad k = 1.3 \times 10^4\, \mathrm{M}^{-1}\mathrm{s}^{-1}$$

R10 $${\text{Cl}}^{\cdot }+{\text{H}}_{2}{\text{O}}_{2}\to {\text{HO}}_{2}^{\cdot }+{\text{Cl}}^{-}+{\text{H}}^{+}\quad k=2.0 \times 10^{9}\;{\text{M}}^{-1} {\text{s}}^{-1}$$

R11 $${\mathrm{Cl}}_{2}^{\cdot }+{\mathrm{H}}_{2}{\mathrm{O}}_{2}\to {\mathrm{HO}}_{2}^{\cdot }+{2\mathrm{Cl}}^{-}+{\mathrm{H}}^{+} \quad k = 1.4 \times 10^{5}\,\mathrm{M}^{-1}\mathrm{s}^{-1}$$

R12 $${{2\mathrm{H}}^{+}+2\mathrm{ClO}}_{3}^{-}+{\mathrm{H}}_{2}{\mathrm{O}}_{2}\to 2{\mathrm{ClO}}_{2}+2{\mathrm{H}}_{2}\mathrm{O}+{\mathrm{O}}_{2}$$

#### KI

**Figure 5:**
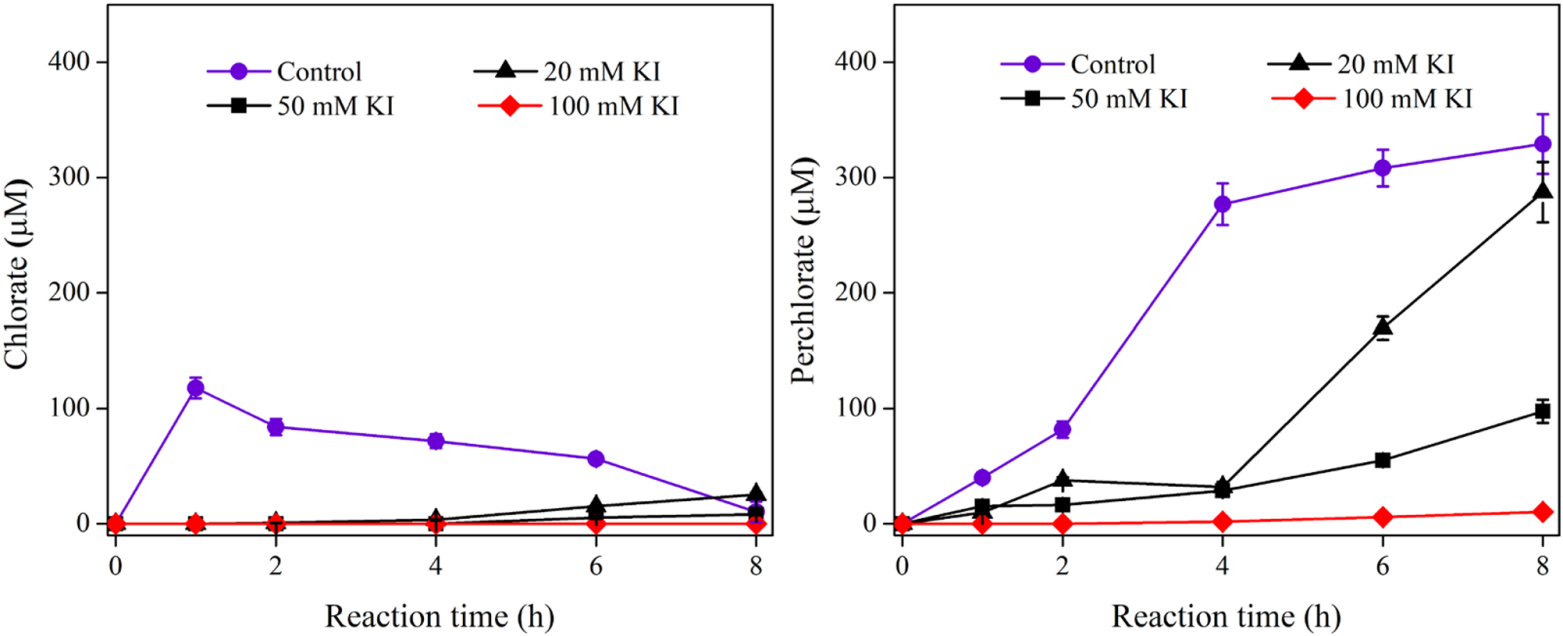
Formation of ClO_3_^−^ and ClO_4_^−^ during the electrochemical oxidation of Cl^−^ in the presence of KI on Ti_4_O_7_ anodes. Conditions: [Cl^−^]_0_ = 1.0 mM, [Na_2_HPO_4_] = 100 mM, current density = 10 mA cm^-2^.

Cl^−^ (Cl^·^/Cl^−^, 2.41 V) and Br^−^(Br^·^/Br^−^, 1.62 V) can be oxidized by HO^·^ to form carcinogenic chlorate and bromate^40^, while I^−^, having a lower reduction potential of 1.33 V^25,41^, may be more readily oxidized than Cl^−^ and Br^−^ in theory^42^. It was also found in our previous studies that NaI may be used as a Cl^−^ free salt to regenerate PFAS-laden ion exchange resin without compromised capability in PFAS recovery^20^. To evaluate the impact of I^−^ on the formation of ClO_3_^−^ and ClO_4_^−^ during EO process, an EO experiment was performed in the presence of I^−^. It should be noted that the anodic potential was relatively constant at the same current density (10 mA cm^2^) with I^−^ at different levels (Fig. S1b). The presence of I^−^ inhibited the formation of ClO_3_^−^ and ClO_4_^−^ significantly as shown in Fig. 5. Almost no ClO_3_^−^ was formed during the first 4.0 h and then increased to 25.5 μM after 8.0 h in the presence of 20-mM KI. Similarly, the formation of ClO_4_^−^ increased slowly during the first 4.0 h and reached 287.2 μM at 8.0 h. Furthermore, near-complete inhibition of ClO_3_^−^ and ClO_4_^−^ formation was achieved when 100 mM KI was spiked, with the values of *k*_*1*_ and *k*_*2*_ decreased to 0 and 4.76 × 10^–6^ s^-1^, respectively (Fig. 4). This suggests that I^−^ outcompetes CI^−^ for reaction with HO^·^, leading to a slower generation of HOCl on Ti_4_O_7_, and thus inhibiting the formation of ClO_3_^−^ and ClO_4_^−^. I^−^ can be oxidized by common oxidants leading to reactive iodine species (e.g., hypoiodous acid (HOI), iodine (I_2_), and iodide radical (I^·^)), and then to iodate (IO_3_^-^), which is not considered carcinogenic because it is rapidly reduced to I^−^ after being ingested^43,44^.

## Conclusions

In conclusion, oxidation of Cl^−^ lead to the formation of ClO_3_^−^ and ClO_4_^−^ on both BDD and Magnéli phase Ti_4_O_7_ anode during EO. This transformation process was much faster on BDD than Ti_4_O_7_ anode in different supporting electrolytes, including Na_2_SO_4_, NaNO_3_, Na_2_B_4_O_7_, and Na_2_HPO_4_. The formation of ClO_3_^−^ and ClO_4_^−^ was easier with Na_2_SO_4_ as supporting electrolyte in both systems. Around 99% and 58% of the total Cl^−^ was transformed to ClO_4_^−^ after 4.0 h of EO with the BDD and Ti_4_O_7_ anode, respectively. When NaNO_3_ was used as electrolytes, ClO_3_^−^ and ClO_4_^−^ formation was decreased to some extent, with only 9% of the total Cl^−^ transformed to ClO_4_^−^ on Ti_4_O_7_ anode. Addition of MeOH, H_2_O_2_, and KI can effectively inhibit the formation of ClO_3_^−^ and ClO_4_^−^ during EO by Ti_4_O_7_ anode. Near complete inhibition of their formation was achieved with 1000-mM MeOH and 100-mM KI present. MeOH, H_2_O_2_, and KI appear to be ideal quenchers to mitigate ClO_3_^−^ and ClO_4_^−^ formation, because they are effective, accessible and inexpensive. In particular, KI is more stable and easier to be stored and transported than MeOH and H_2_O_2_. I^−^ is oxidized to iodate ultimately in the EO system, while iodate is a relatively stable and benign chemical. In practice, the EO treatment can be designed to fully convert I^−^, or else a polishing step, such as IXR, has to be followed to remove remaining I^−^. The findings provide a basis for devising strategies to reduce the formation of ClO_3_^−^ and ClO_4_^−^ in the EO process.

## Supplementary Information

Supplementary Information.

## Data Availability

The SEM collected during the current study is available in the NoMad repository, 8uLLHSolQ06aHg2roegwpg.
