# Supplementary material for: Formation of chlorate and perchlorate during electrochemical oxidation by Magnéli phase Ti4O7 anode: inhibitory effects of coexisting constituents
Source: Sci Rep. 2022 Sep 23;12:15880. doi: 10.1038/s41598-022-19310-5 (PMC9508142; doi:10.1038/s41598-022-19310-5)
Supplement: Supplementary file 1 — Supplementary Information. [file 41598_2022_19310_MOESM1_ESM.docx]

**Supporting Information**

# Formation of Chlorate and Perchlorate during Electrochemical Oxidation by Magnéli Phase Ti_4_O_7_ Anode: Inhibitory Effects of Coexisting Constituents

Lu Wang^a, b^, Yaye Wang^c^, Yufei Sui^c^, Junhe Lu^b^, Baowei Hu^a^, Qingguo Huang^c,^ *

^a^ School of Life Science, Shaoxing University, Shaoxing, 312000, China

^b^ Department of Environmental Science and Engineering, Nanjing Agricultural University, Nanjing, 210095, China

^c^ Department of Crop and Soil Sciences, University of Georgia, Griffin, GA 30223, USA

*Corresponding author:

E-mail: [qhuang@uga.edu](mailto:qhuang@uga.edu) Telephone: +1-770-2293302

2 texts, 3 figures

**Text S1**

## Electrode fabrication and characterization

The Magnéli phase Ti_4_O_7_ electrode was fabricated through a high temperature sintering process as described in our previous study (H. Lin et al. Chemical Engineering Journal, 2018, 354,1058-1067). In brief, TiO_2_ powder was heated at 950 ^◦^C and reduced to Ti_4_O_7_ powder under controlled H_2_ flow. The Ti_4_O_7_ powder was mixed (0.5%, m/m) with polyacrylamide/polyvinyl alcohol (95/5, m/m) to form a slurry that was then spray-dried to form ceramic granulates. The ceramic granulates were then pressed to make a ceramic preform that was dried and then sintered at 1350 °C in a vacuum for 11 h to form a bulk electrode.

The chemical composition of the Magnéli phase Ti_4_O_7_ electrode was characterized using an X’Pert PRO MRD X-ray diffractometer (XRD) (PANalytical, Netherlands) with CuKα1 radiation at 40 kV/40 mA. The surface morphology of Ti_4_O_7_ electrode was examined using scanning electron microscopy (SEM) on a Hitachi’s-4800 FE-SEM system (Hitachi, Japan). The porosimetry analysis of the anode was measured using a Micromeritics Autopore IV 9500 mercury porosimeter (Norcross, GA) according to method ISO 15901-1.

The XRD pattern confirmed Magnéli phase Ti_4_O_7_ as the dominant composition of the electrode material (Fig. S3 A and B). The SEM image (Fig. S3 C) shows fairly uniform interconnecting pores with diameters smaller than 10 µm on the surface of Magnéli phase Ti_4_O_7_ anode material. Mercury intrusion porosimetry analysis (Fig. S3 D) indicates a porosity of 21.6%, and a median pore diameter of 3.6 μm (based on volume) or 2.8 μm (based on area), and an average pore diameter of 2.6 μm.

**Text S2**

**UPLC-MS/MS Analysis**

Formation of ClO_3_^−^ and ClO_4_^−^ were quantified using the ultra-high performance liquid chromatography and mass spectrometry (UPLC-MS/MS, Waters ACQUITY, Milford, MA). An ACQUITY UPLC BEH 1.7-micron C18 column (Waters, Milford, MA) was used for UPLC separation. The mobile phase consists of 50% water (A) and 50% MeOH (B) running at 0.3 mL min^−1^. Electrospray ionization was operated in negative mode with the capillary voltage at 3 kV and the source temperature at 400 °C. ClO_3_^−^ and ClO_4_^−^ were quantified using multiple reactions monitoring (MRM) based on the transition m/z = 83 > 67 for ClO_3_^−^, and m/z = 99 > 83 for ClO_4_^−^. Quantification was achieved using a five-point calibration curve. The detection limits for ClO_3_^−^ and ClO_4_^−^ were 50.0 μg L^−1^ and 25.0 μg L^−1^, respectively, with an injection volume of 10.0 μL.

**



**

**Fig. S1** The anodic potential on Ti_4_O_7_ in Na_2_SO_4_ solution with MeOH (a) and KI (b) spiked during the oxidation of Cl^−^. [Cl^−^]_0_= 1 mM, [Na_2_HPO_4_]= 100 mM, current density = 10 mA‧cm^-2^.







**Fig. S2** Formation of ClO_3_^−^ and ClO_4_^−^ during the electrochemical oxidation of Cl^−^ in the presence of H_2_O_2_ on Ti_4_O_7_ anodes. Conditions: [Cl^−^]_0_= 1 mM, [Na_2_HPO_4_] = 100 mM, current density = 10 mA‧cm^-2^.


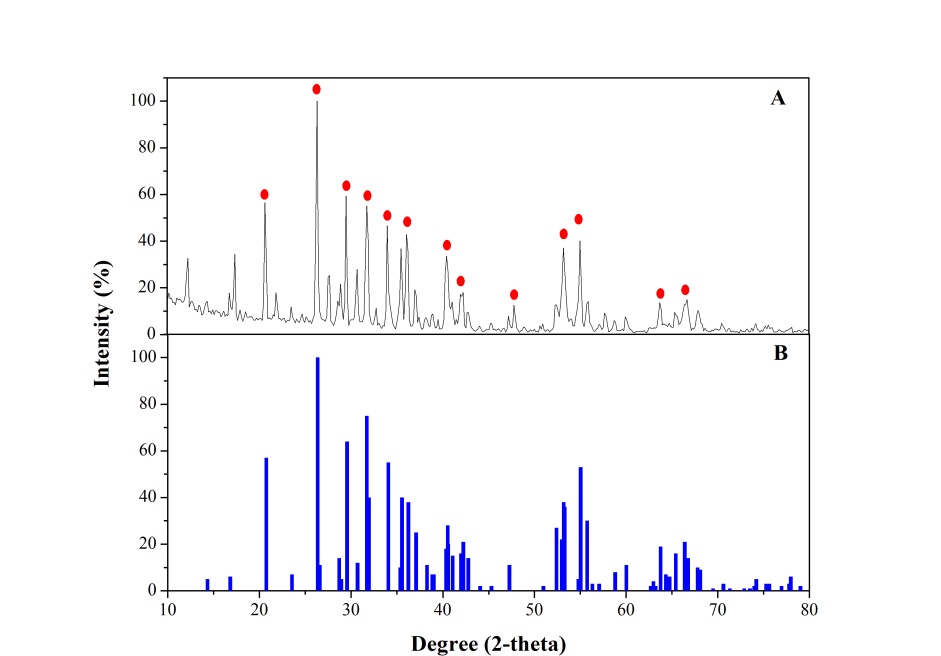


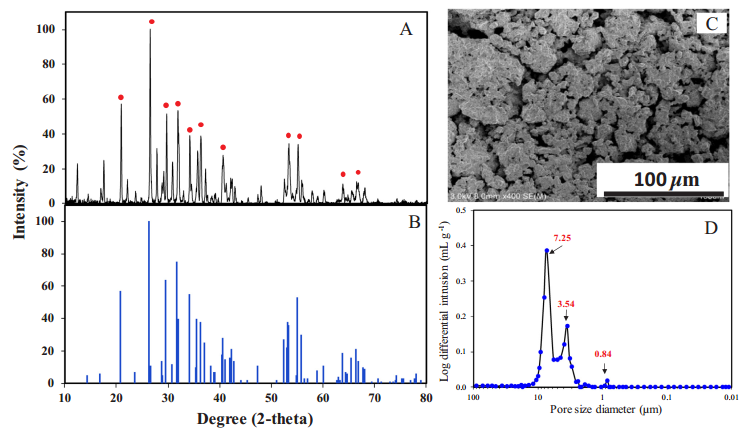


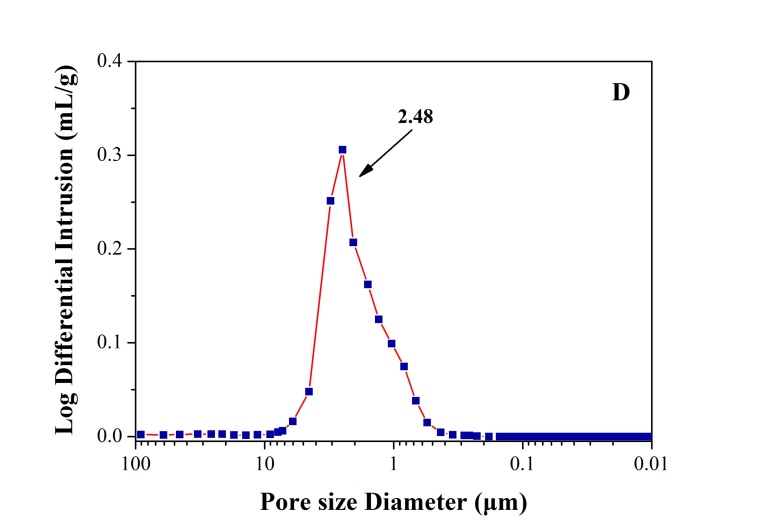


**Fig. S3** XRD data of Magnéli phase Ti_4_O_7_ electrode material (A) and reference Ti_4_O_7_ powder (B); SEM image of Ti_4_O_7_ electrode material (C); Result of mercury intrusion analysis of pore size distribution (D).
